# ASO Practice Guidelines Series: Surgical Management of Bladder Cancer Relapse

**DOI:** 10.1245/s10434-026-19129-8

**Published:** 2026-02-10

**Authors:** Can Aydogdu, Sean T. McSweeney, Vignesh T. Packiam, Laura Bukavina

**Affiliations:** 1 Department of Urology, Glickman Urological and Kidney Institute, Cleveland Clinic, Cleveland, OH USA; 2 Section of Urologic Oncology, Rutgers Cancer Institute, New Brunswick, NJ USA

## Abstract

**Background:**

When bladder cancer recurs after bacillus Calmette–Guérin (BCG) for high-risk non-muscle-invasive disease, after trimodal therapy (TMT), or after other bladder-preserving therapy for muscle-invasive disease, patients face time-sensitive decisions that determine whether cure remains achievable. Treating physicians must guide these choices by balancing oncologic safety with quality of life, comorbidities, and patient preferences.

**Methods:**

We reviewed 2024–2025 guidelines from the American Urological Association/Society of Urologic Oncology (AUA/SUO), National Comprehensive Cancer Network (NCCN), and European Association of Urology (EAU), while incorporating pivotal trials and recent drug approvals. Emphasis was placed on translating recommendations into practical decision-making for clinicians and patients.

**Results:**

For post-BCG recurrence, early radical cystectomy (RC) offers the highest chance of cure for medically fit patients and is recommended throughout all three guidelines. When surgery is not possible or declined, bladder-sparing therapies, including four recently US Food and Drug Administration (FDA)-approved drugs, can be considered with strict surveillance. In post-TMT recurrence, RC is standard for invasive relapse, with bladder preservation reserved for select noninvasive cases. In both settings, optimal outcomes require timely workup, presentation of all viable options, and coordination of multidisciplinary input with a strong emphasis on close surveillance and follow-up.

**Conclusions:**

Management of bladder cancer recurrence is optimized when therapy aligns with patient goals while safeguarding oncologic outcomes. Regardless of the path chosen, early engagement of a multidisciplinary team and shared decision-making are essential to delivering the best possible care.

Recurrence after initial bladder-preserving therapy is a complex challenge in the management of bladder cancer. In patients with high-risk non-muscle-invasive bladder cancer (HR-NMIBC), intravesical bacillus Calmette–Guérin (BCG) therapy remains the standard of care, yet treatment failure is common. Up to 30–40% of patients do not respond to BCG, and among those with persistent or recurrent high-grade disease, the risk of progression to muscle-invasive bladder cancer may approach 50%. These outcomes, particularly in the setting of BCG-unresponsive disease, highlight the aggressive biology of HR-NMIBC and the urgent need for effective alternative treatment strategies.^1^

In muscle-invasive bladder cancer (MIBC), recurrence after trimodality therapy (TMT) or other bladder-preserving strategies occurs less often but is clinically significant. Long-term series in noninvasive disease (Tis, Ta, T1) report any intravesical recurrence in roughly 25–30% of patients, with T2 muscle-invasive relapse in 10–15%. Such failures often reflect aggressive tumor biology and carry a substantial risk of metastasis if not addressed promptly.^2–6^

For patients, these scenarios bring time-sensitive decisions that may determine whether cure remains possible. For treating physicians, they demand rapid assessment, clear explanation of options, and alignment of treatment with oncologic safety, comorbidities, and patient priorities. Delays can close the window for curative intervention, particularly in BCG-unresponsive disease or invasive relapse post-TMT.

Radical cystectomy (RC) remains the most effective intervention for long-term cancer control when the patient is medically fit for surgery.^7–11^ However, not all patients are surgical candidates, and many seek to avoid surgery owing to its impact on urinary function, body image, and quality of life. Recent advances in bladder-sparing therapies, such as systemic immunotherapy, gene therapy, and novel intravesical drug-delivery systems, offer additional options for selected patients but require strict surveillance and timely salvage when failure occurs.

This review synthesizes current evidence and guidelines for recurrence after BCG and after TMT or other bladder-preserving therapies, focusing on patient-centered pathways where physician expertise and multidisciplinary collaboration optimize both oncologic and quality-of-life outcomes.

## Management of Relapse After BCG

Overall, non-muscle-invasive bladder cancer (NMIBC) accounts for about 75% of all bladder cancer cases.^12^ Treatment strategies include surveillance post transurethral resection of bladder tumor (TURBT), adjuvant intravesical chemotherapy or immunotherapy, and early radical cystectomy in a subset of very high-risk patients. In clinical series, 20–40% of NMIBC patients recur after intravesical BCG treatment, and approximately 30% of these BCG failures demonstrate pathological progression to ≥ T2 disease at cystectomy.^7,10,13^ Importantly, there has been recent attention and approval of novel pharmacologic agents and treatment strategies tailored to this indication.

### Definition of BCG Failure

To accurately classify patients who are unlikely to benefit from further BCG after treatment failure, a variety of terms and definitions have been introduced. Among these, the US Food and Drug Administration (FDA) definition of “BCG-unresponsive” is the most widely recognized, having been adopted by all major North American guidelines.^14^ This definition was initially established to provide a standardized framework for clinical trial design in the setting of BCG failure. Table 1 provides a side-by-side comparison of North American and European definitions. Importantly, there is consensus from both regions on what constitutes adequate BCG therapy. Similarly, the definitions of BCG-unresponsive disease are largely aligned, although the EAU guidelines include additional scenarios classified under BCG-refractory disease. Furthermore, the EAU offers specific definitions for BCG-relapsing, BCG-exposed, and BCG-intolerant disease.^7^

**Table 1:** Comparison of definitions of BCG-unresponsive definitions across guidelines

**FDA**^14^		**EAU 2025**^7^	
≥ 5 of 6 doses iBCG **+** ≥ 2 of 3 doses mBCG **or** ≥ 2 of 6 doses re-iBCG	**Adequate BCG**	≥ 5 of 6 doses iBCG **+** ≥ 2 of 3 doses mBCG **or** ≥ 2 of 6 doses re-iBCG	
**Persistent** or **recurrent** CIS ± Ta/T1 within 12 months of completion of adequate BCG	**BCG-unresponsive**	**Persistent** or **recurrent** CIS ± Ta/T1within 12 months of completion of adequate BCG	
**Recurrent** Ta/T1 HG within 6 months of completion of adequate BCG		**Recurrent** Ta/T1 HG within 6 months of completion of adequate BCG	
**Presence** of T1 HG after iBCG at first evaluation		**Presence** of T1 HG at 3 months	**BCG-refractory**
		**Presence** of any HG tumor during mBCG	
		**Presence** of Ta HG at 3 **and/or** 6 months after re-iBCG or mBCG	
		**Presence** of CIS at 3 months **and persistence** at 6 months after re-iBCG or mBCG	
		**Recurrence** of HG tumor after completion of mBCG, despite initial response	**BCG-relapse**
		**Presence** of Ta HG **or** CIS at 3 months after iBCG only OR **delayed relapse** after adequate or inadequate BCG	**BCG-exposed**
		Severe side effects that prevent further BCG instillation before completing treatment	**BCG-intolerant**
**Recurrence** of LG tumor during or after BCG treatment	**Not BCG-unresponsive**	**Recurrence** of LG tumor during or after BCG treatment	

The bolding is to draw attention to differences and the specific catetogires that are present in Table 1

*BCG* bacillus Calmette–Guérin, *iBCG* BCG induction, *mBCG* BCG maintenance, *re-iBCG* BCG reinduction, *CIS* carcinoma in situ, *HG* high-grade, *LG* low-grade, *Ta* papillary tumor, *T1* papillary tumor invading the lamina propria

It should be emphasized that not every recurrence after adequate BCG qualifies as BCG-unresponsive disease. In particular, all low-grade recurrences are excluded from this category and should not be considered BCG-unresponsive.^7,14^

### Follow-Up Regimens during/post-BCG

Follow-up in NMIBC is risk-stratified across all major international guidelines. Most patients treated with BCG, including those with BCG-unresponsive disease, are classified as high or very high risk. While the definitions of risk categories differ somewhat between the NCCN/AUA/SUO and EAU guidelines, follow-up recommendations for these risk groups are broadly aligned. Surveillance generally consists of regular cystoscopy, urine cytology, and imaging including upper tract evaluation, as summarized in Table 2. A notable distinction is that the EAU guidelines explicitly recommend lifelong follow-up, whereas the NCCN and AUA/SUO guidelines do not define a fixed duration, leaving the length of surveillance to physician judgment and shared decision-making with the patient.^7,10,11^

**Table 2:** Guidelines recommendations for follow up procedures and tests for high-risk NMIBC, which includes BCG-unresponsive disease

Years		AUA/SUO 2024^10^	NCCN 2025^11^	EAU^1^ 2025^7^
1–2	Cystoscopy	q3–4m	q3m	q3m
	Urine cytology	q3–4m	q3m	q3m
	Imaging	UUT^2^ q1–2y	UUT^2^ baseline + 12m, then q1–2y	CTU q1y
3–4	Cystoscopy	q6m	q6m	q6m
	Urine cytology	q6m	q6m	q6m
	Imaging	UUT^2^ q1–2y	UUT^2^ q1–2y, CT A/P as clinically indicated	CTU q1y
5	Cystoscopy	q1y	q6m	q6m
	Urine cytology	q1y	q6m	q6m
	Imaging	UUT^2^ q1–2y	UUT^2^ q1–2y, CT A/P as clinically indicated	CTU q1y
6–10	Cystoscopy	q1y	q6m	q1y
	Urine cytology	q1y	q6m	q1y
	Imaging	UUT^2^ q1–2y	UUT^2^ q1–2y, CT A/P as clinically indicated	CTU q2y
> 10	Cystoscopy	^3^	As clinically indicated	q1y
	Urine cytology		As clinically indicated	q1y
	Imaging		As clinically indicated	^4^

*UUT* upper urinary tract imaging, *CT A/P* CT of the abdomen and pelvis, *CTU* CT urography, *q3m/q6m/q1y/q2y* every 3 months/every 6 months/every 1 year/every 2 years

^1^EAU guideline recommends life-long follow-up

^2^Both AUA/SUO and NCCN recommend CTU for UUT imaging, with MR urography as an alternative if CTU is not feasible

^3^AUA/SUO guidelines do not elaborate on a follow-up schedule beyond 10 years

^4^EAU guideline state imaging follow-up for up to 10 years

### Guideline-Based Management for BCG-Unresponsive Patients (AUA, NCCN, and ASCO)

Across all major guidelines (AUA, NCCN, ASCO, and EAU), radical cystectomy remains the recommended standard of care for patients with BCG-unresponsive disease who are fit for surgery. For those unwilling or medically ineligible, enrollment in clinical trials is strongly advised, with bladder-sparing strategies reserved for situations where trial participation is not feasible.^7,10^ Key recommendations from the guidelines are summarized in Table 3, while recently approved intravesical and systemic therapies that have broadened treatment options including efficacy data are outlined in Table 4. Given the biological heterogeneity of BCG-unresponsive disease, management should be individualized on the basis of disease features, comorbidities, and patient preferences. Despite the curative potential of radical cystectomy, real-world data show that many patients decline surgery, underscoring the need for effective and durable bladder-sparing strategies.^15^

**Table 3:** Comparison of guidelines statements for treatment of patients with BCG-unresponsive disease

	AUA/SUO 2024^10^	NCCN 2025^11^	EAU 2025^7^
Eligible for RC	RC (**moderate recommendation, evidence strength grade C**)	RC (**preferred**)	Offer RC (**Strong recommendation)**
Ineligible or refuse RC	Clinical trial enrollment Alternative intravesical therapy (i.e., nadofaragene firadenovec) Alternative intravesical chemotherapy (i.e. Gemcitabine + Docetaxel, Valrubicin) Systemic Pembrolizumab (CIS within 12 months of completion of adequate BCG)	Intravesical chemotherapy Pembrolizumab CIS ±Ta/T1 (**category 2A**) HG Ta/T1 NMIBC only (**category 2b**) Nadofaragene firadenovec CIS ±Ta/T1 (**category 2A**) HG Ta/T1 NMIBC only (**category 2b**) N803+BCG for CIS ± Ta/T1 (**category 2A**)	Any of the following (preferred within in clinical trial setting): Intravesical chemotherapy Chemotherapy and microwave-induced hyperthermia Electromotive administration of chemotherapy Intravesical- or systemic immunotherapy **(Weak recommendation)**

Bold is in place to highlight important information such as the level of recommendation

*RC* radical cystectomy, *CIS* carcinoma in situ, *HG* high-grade, *Ta/T1* papillary tumor, *BCG* bacillus Calmette–Guérin

**Table 4:** Key efficacy outcomes of emerging agents in the BCG-unresponsive setting, including complete response rates, durability of response, disease-free survival, safety, and regulatory status. Regulatory approval status as of September 2025

		Pembrolizumab^61,62^	Nadofaragene firadenovec^63^	N-803 + BCG^64^	TAR-200^65^	Cretostimogene grenadenorepvec ^66^
Reinduction		no	yes	yes	no	yes
CIS ± Ta/T1 (*n*)		96	103	82	85	110
CR at 3 months		41%	53%	55%	79%	75%^1^
CR at 12 months		19%	24%	45%	46%	46%
mDoR		16.2 mo	9.7 mo	26.6 mo	25.8 mo	27.9 mo
Ta/T1 only (*n*)		132	48	72	52	75 (planed)^2^
DFS at 12 months		44%	44%	55%	70%	^2^
mDFS		7.7 mo	12.4 mo	19.3 mo	NR^3^	^2^
Grade ≥ 3 AEs		13–15%	4%	23%	14%	0%
FDA	CIS ± Ta/T1	yes^30^	yes^31^	yes^32^	yes^33^	no
	Ta/T1 only	no^3^	no^3^	no	no	no
EMA		no	no	pending	no	no

*CIS* carcinoma in situ, *CR* complete response, *m* median, *mo* months, *DOR* duration of response, *Ta/T1* papillary tumor, *DFS* disease-free survival, *AE* adverse event, *FDA* US Food and Drug Administration, *EMA* European Medicines Agency

^1^Response at any time; no 3-month CR data available

^2^No data available at time of manuscript preparation or not reached at data cutoff

^3^Category 2B recommendation by NCCN

Emerging evidence from several retrospective series suggests that an initial bladder-sparing approach can be oncologically safe in carefully selected patients with BCG-unresponsive high-grade NMIBC.^16,17^ Across multi-institutional cohorts, patients managed with intravesical or systemic salvage therapy showed no meaningful difference in cancer-specific or metastasis-free survival compared with those undergoing upfront radical cystectomy.^16,17^ In one analysis, individuals receiving salvage intravesical therapy before eventual cystectomy demonstrated similar rates of pathological upstaging and 5-year bladder cancer-specific survival (73–74%) to immediate surgery.^17^ An international cohort, in which most patients initially chose bladder preservation, likewise reported comparable intermediate-term cancer-specific survival, although high-grade recurrences remained common (37% at 1 year and 52% at 2 years) and progression to muscle-invasive disease occurred in a minority (13% at 2 years).^18^ Most recurrences were amenable to additional intravesical therapy or timely deferred cystectomy, and approximately one-third of patients ultimately underwent salvage cystectomy, typically with favorable pathological outcomes.^17,18^ Appropriate selection remains critical, as candidates for bladder preservation generally lack adverse pathological features and have disease suitable for complete endoscopic resection.^16^ Long-term data also support this approach, with sequential intravesical chemotherapy achieving 5-year progression-free and cancer-specific survival rates of approximately 81% and 88%, respectively, and durable bladder preservation in most patients.^19^ Taken together, these findings indicate that an initial bladder-sparing strategy, combined with rigorous surveillance, represents a feasible alternative to immediate cystectomy in selected patients with BCG-unresponsive NMIBC.^16,19^

The prospective multicenter CISTO study (NCT03933826) was designed to address an important evidence gap by systematically comparing patient-reported and patient-centered clinical outcomes in individuals with recurrent high-grade NMIBC who elect for either a bladder-sparing approach or radical cystectomy. The CISTO study demonstrated that physical function scores at 12 months were similar between groups, while several secondary measures, including global health, anxiety, depression, and financial well-being, were more favorable among patients who underwent radical cystectomy.^20,21^ The study remains ongoing, and longer-term follow up will be essential to clarify whether these differences persist and to define longer-range quality-of-life and clinical outcomes.

#### Intravesical Chemotherapy

Current guidelines do not provide specific recommendations for particular intravesical chemotherapy regimens. Valrubicin received FDA approval in 1998 for BCG-refractory carcinoma in situ (CIS), but its use has remained limited owing to poor durable efficacy.^22–24^

In recent years, intravesical gemcitabine/docetaxel (Gem/Doce) combination therapy has gained increasing popularity among US physicians, despite the absence of prospective data in the BCG-unresponsive setting.^25^ According to the original 2015 Gem/Doce study, this regimen includes six weekly instillations of 1 g of gemcitabine followed by 37.5 mg of docetaxel.^26^ In a multi-institutional retrospective analysis of 276 patients with recurrent NMIBC previously treated with BCG, high-grade recurrence-free survival was 65% at 1year and 52% at 2 years, with disease progression occurring in only 4% of patients.^25^ A separate longer-term follow-up study of 97 patients reported high-grade recurrence-free survival rates of 60%, 50%, and 30% at 1, 2, and 5 years, respectively, with no grade 3 or higher adverse events.^27^ Among those with BCG-unresponsive disease, 5 year outcomes showed recurrence-free survival of 28%, progression-free survival of 89%, cystectomy-free survival of 74%, and overall survival of 66%, indicating that most recurrences can be managed safely without progression or the need for cystectomy. Despite the mentioned lack of prospective data in the BCG-unresponsive setting, Gem/Doce is also starting to get used in Europe.^28^

The phase 3 BRIDGE trial (NCT05538663) is currently ongoing to evaluate this regimen in the BCG-naïve population.^29^

#### Pembrolizumab

The programmed cell death protein 1 (PD-1) inhibitor pembrolizumab is administered intravenously every 3 weeks for up to 35 cycles. In January 2020, the US FDA approved pembrolizumab for patients with BCG-unresponsive CIS with or without papillary disease who are ineligible for or declined radical cystectomy (see Table 4 for efficacy data).^30^

Can you add a couple sentences of safety/efficacy data from the trial. And comment that this is infrequently utilized due to that profile and concern for rare but permanent high grade AEs.

According to the NCCN guidelines, pembrolizumab is listed as a category 2A recommendation for CIS and category 2B for papillary-only disease.^11^ The AUA guidelines state that it may be offered to patients with CIS recurrence within 12 months after completion of adequate BCG therapy who are declining or ineligible for radical cystectomy.^10^ In contrast, the EAU guidelines recommend that systemic immunotherapy should only be administered within clinical trials in this setting.^7^

#### Nadafaragene Firadenovec

Nadofaragene firadenovec is a nonreplicating adenoviral vector-based intravesical gene therapy that delivers the interferon alfa-2b gene to urothelial cells, leading to sustained local production of interferon. It is instilled intravesically via a Foley catheter once every 3 months. The FDA approved nadofaragene firadenovec in December 2022 for adults with BCG-unresponsive high-risk non-muscle-invasive bladder cancer with CIS with or without papillary tumors who are ineligible for or decline radical cystectomy.^31^ Key efficacy results from this pivotal trial are summarized in Table 4.

Because its approval predates the most recent guideline updates, recommendations are still limited. While the NCCN now lists nadofaragene firadenovec as a treatment option for BCG-unresponsive CIS ± papillary disease (category 2A), as well as papillary disease alone (category 2B), the AUA and EAU guidelines have yet to fully incorporate it into their treatment algorithms. As of now, there is no European Medicines Agency (EMA) approval.^7,10,11^

#### N803+BCG

Nogapendekin alfa inbakicept-pmln (N-803; Anktiva) is an interleukin (IL)-15 superagonist administered intravesically in combination with BCG. Treatment consists of a 6 week weekly induction course, followed by 3 week maintenance courses given weekly at months 4, 7, 10, 13, and 19. For patients with ongoing response, additional maintenance courses may be administered at months 25, 31, and 37.^32^

In April 2024, the US FDA approved N-803 plus BCG for patients with BCG-unresponsive CIS with or without papillary tumors, with efficacy data of the registrational trial displayed in Table 4.^32^ This regimen is also recommended in the NCCN guidelines (category 2A).^11^ Currently, N-803 plus BCG for BCG-unresponsive CIS ± papillary disease is under review by the EMA.

#### TAR 200

TAR 200 is a novel intravesical drug-delivery device that releases a continuous gemcitabine dose within the bladder. This device is inserted via Foley catheter and removed cystoscopically every 3 weeks for up to eight doses, followed by maintenance applications every 12 weeks for an additional 18 months. It received FDA approval in September 2025 (see Table 4 for efficacy data).^33^ As the approval is very recent, there are no recommendations yet in current clinical guidelines, except for the latest NCCN guideline which includes a category 2A recommendation.

### Investigational Drugs for BCG-Unresponsive NMIBC

#### Cretostimogene Grenadenorepvec

This is an oncolytic adenovirus engineered to selectively replicate in tumor cells with defective retinoblastoma (Rb) pathways and to deliver granulocyte–macrophage colony-stimulating factor (GM-CSF), thereby stimulating local antitumor immunity. It is administered intravesically by Foley catheter once weekly for 6 weeks (induction), followed by maintenance dosing every 3 months for up to 1 year, and every 6 months in the second year.

Cretostimogene is currently an investigational therapy and has not yet received FDA or EMA approval. It has been granted Breakthrough Therapy and Fast Track designations by the FDA for patients with BCG-unresponsive NMIBC with CIS with or without papillary tumors. In the phase 3 BOND-003 trial (NCT04452591), cretostimogene demonstrated a high initial complete response rate with durable responses at 12 months, but regulatory submissions are still pending (see Table 4 for efficacy data). Accordingly, there are no recommendations yet reflected in the current guidelines.

#### Other Drugs under Investigation

A number of additional agents are in active clinical development for high-risk NMIBC. For most trials reported hereafter, data from small preliminary cohorts exist and are reported at scientific conferences. Owing to the lack of proper publications, we do not report oncologic outcomes at this time.

Pembrolizumab combined with BCG is being evaluated in the phase 3 KEYNOTE-676 trial, which compares the combination with BCG alone in patients with persistent or recurrent high-risk disease (NCT03711032).^34^

TAR210 is a novel intravesical drug-delivery system (similar to TAR200) that provides continuous local release of the fibroblast growth factor receptor (FGFR) inhibitor erdafitinib into the bladder. It is being studied in patients with FGFR-altered NMIBC in the Moonrise-3 trial (NCT06919965).

TARA-002 is an intravesical immunotherapeutic derived from a genetically modified *Streptococcus pyogenes* preparation that activates both innate and adaptive antitumor responses. It is currently under evaluation in the ADVANCE phase 2 study, which includes both BCG-naïve and BCG-unresponsive cohorts with CIS ± Ta/T1 (NCT05951179).

EG-70 (detalimogene voraplasmid) is a nonviral intravesical gene therapy that delivers plasmids encoding IL-12 together with retinoic acid-inducible gene (RIG)-I stimulatory elements to induce a robust local immune response. It is being assessed in the ongoing phase 1/2 LEGEND trial in BCG-naïve and BCG-unresponsive patients (NCT04752722).

NDV-01 (Gem/Doce) is a sustained-release intravesical formulation of gemcitabine and docetaxel embedded within a bioadhesive gel matrix that maintains prolonged drug exposure in the bladder for approximately 10 days. It is under investigation in a phase 2 study enrolling patients with high-grade NMIBC, including those with high-grade recurrence after BCG induction (NCT06663137).

## Management of Relapse after Bladder Preserving Therapy

### Definition of Failure of Trimodal Therapy

**Fig. 1:**
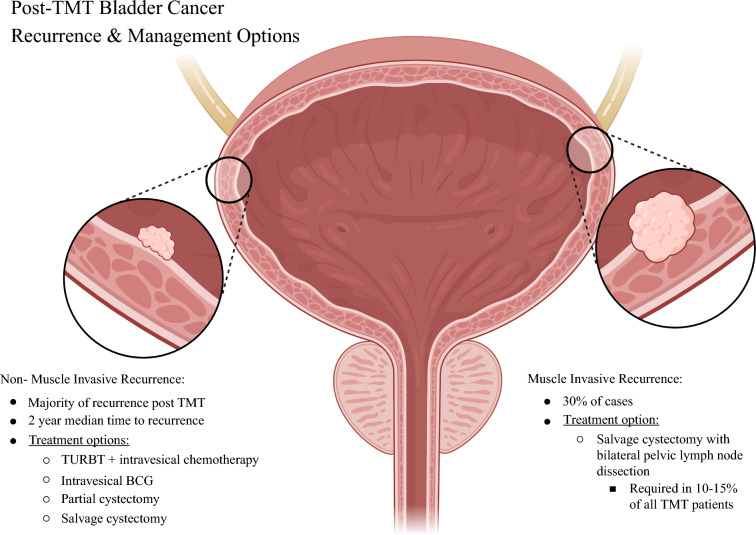
Patterns of recurrence and management after trimodal therapy (TMT) for bladder cancer. Most recurrences are non-muscle-invasive and occur at a median of 2 years, managed with TURBT, intravesical therapy, or salvage surgery. About 30% are muscle invasive, requiring salvage cystectomy with bilateral pelvic lymph node dissection in 10–15% of all TMT patients. Metastatic recurrence (not shown) is managed with systemic therapy

With advances in systemic therapy, many patients now achieve complete clinical remission after neoadjuvant chemotherapy.^35^ As a result, both patients and physicians increasingly question the necessity of upfront radical cystectomy, given its significant impact on quality of life, as well as physiological, psychological, and sexual function. Bladder-preserving approaches for MIBC include trimodal therapy (TMT), partial cystectomy, radiation therapy, or systemic therapy alone (Fig. 1).

Ideally, patients achieve long-term complete clinical remissions after curative-intent bladder-preserving therapy. As a part of TMT, the patient undergoes upfront maximal TURBT, which has been shown to increase local tumor control by up to 20%.^36,37^ Prior to proceeding with consolidative external beam radiation therapy (XRT), patients should undergo restaging TURBT to assess for progression of invasive (T2) disease, prompting discussion regarding radical cystectomy. Patients without invasive disease then complete XRT and radiosensitizing chemotherapy and are assessed for response following treatment. Repeat TURBT has been shown to be positive for residual tumor in up to 50% of patients, highlighting the importance of maximal initial resection, and also the necessity for restaging TURBT.^38^

Approximately 30% of these patients who undergo TMT will have a locally muscle-invasive recurrence. Additionally, patient-reported outcomes have been shown to be similar if not better in RC patients versus those undergoing TMT, highlighting the continued role for RC in patients with recurrent HR-NMIBC in this setting.^21^

Patients found to have distant metastatic disease (M1) following TMT should be referred to medical oncology and initiated on systemic therapy for metastatic urothelial carcinoma (mUC). Currently, the NCCN-preferred regimen is the combination of the antibody–drug conjugate enfortumab-vedotin and the immune checkpoint inhibitor pembrolizumab. This regimen has dramatically changed the treatment landscape for mUC and is changing the longstanding paradigm of first-line cisplatin-based chemotherapy.^21^

### Follow-Up Regimens during and after TMT/Bladder-Preserving Therapy

Following completion of TMT or bladder-preserving therapy, the AUA, NCCN, and EAU all recommend following the patient with routine cystoscopy, cytology, laboratory work, and CT imaging. Close follow-up is critical in these patients to assess for TMT treatment relapse and need for further therapy. The surveillance protocols for each society are summarized in Table 5.

**Table 5:** Recommended surveillance after curative-intent bladder-sparing trimodality therapy, including cystoscopy, urine cytology, and cross-sectional imaging with abdominal and chest evaluation

Years		AUA/SUO 2024^9^	NCCN 2025^11^	EAU^1^ 2025^8^
1–2	Cystoscopy	q3m	q3m	q3–4m
	Urine cytology	Recommended*	q6–12m	q3–4m
	CT/MRI abdomen^1,2^	q6m	q3–6m	q3–4m
	CT chest	q6m	q3–6m	q3–4m
3	Cystoscopy	q6–12m	q6m	q3–4m
	Urine cytology	Recommended*	as clinically indicated	q3–4m
	CT/MRI abdomen^1,2^	q1y	q1y	q6–12m
	CT chest	q1y	q1y	q6–12m
4	Cystoscopy	q6–12m	q6m	q6m
	Urine cytology	as clinically indicated	as clinically indicated	q6m
	CT/MRI abdomen^1,2^	q1y	q1y	q6–12m
	CT chest	q1y	q1y	q6–12m
> 5	Cystoscopy	q6–12m	q6m	q6m
	Urine cytology	as clinically indicated	as clinically indicated	q6m
	CT/MRI abdomen^1,2^	q1y	as clinically indicated	as clinically indicated
	CT chest	q1y	as clinically indicated	as clinically indicated

*q3m/q6m/q1y* every 3 months/every 6 months/every 1 year

^1^NCCN guidelines recommend CTU/MRU for abdominal imaging

^2^NCCN guidelines recommend FDG PET/CT if metastatic disease suspected

^*^AUA guidelines recommend cytology, however no specific times given

### Guideline-Based Management (AUA, NCCN, and ASCO)

The majority of relapses following TMT or bladder-sparing surgeries are noninvasive. Overall, recurrence rates after initial treatment are around 25%.^2^ Most of these recurrences are noninvasive, at approximately 70%. The median time to recurrence of noninvasive disease following TMT is 2 years, and most muscle invasive local recurrence and metastatic recurrence is found within 5 years.^39^

Those patients found to have relapse of non-muscle-invasive disease are often treated as having de novo NMIBC. They may be offered similar treatment options such as TURBT with intravesical chemotherapy, partial cystectomy, or radical cystectomy with pelvic lymph node dissection depending on the location and burden of disease. It is important to note, however, that non-muscle-invasive relapse following TMT predicts subsequent relapse of both invasive and noninvasive disease, with CIS accounting for 67% of noninvasive relapse, potentially requiring subsequent RC.^39^ Additionally, it is critical to mention the importance of close and prolonged follow-up for TMT patients, as relapses have been reported in patients up to 10 years following their initial TMT.^2^ Noninvasive recurrence is associated with improved disease-specific survival and has been shown to be responsive to BCG. In one series, 59% of patients with noninvasive recurrence treated with induction and maintenance BCG experienced no further recurrence of any kind.^40^

Those patients found to have relapse with muscle-invasive disease should be counseled on salvage radical cystectomy. Current AUA and EAU guidelines recommend radical cystectomy with bilateral pelvic lymph node dissection for TMT or bladder-sparing patients with muscle-invasive recurrence, for those medically fit for surgery. Guidelines also stress the need to counsel patients regarding the increased morbidity with salvage cystectomy, compared with up-front initial cystectomy. Salvage cystectomy is required in only around 10–15% of TMT patients. However, one series examining 118 patients showed that prior TMT does confer an almost twofold higher risk of death compared with upfront RC (hazard ratio (HR) 1.9). Post-TMT patients requiring salvage cystectomy were also shown to have higher odds of postoperative admission to the intensive care unit (ICU) (odds ratio (OR) 2.8) with increased operative time, intraoperative blood loss, as well as 30 and 90-day perioperative complications (bowel injury, anastomotic stricture, etc.) compared with primary RC patients.^41^ Another study showed similar results with higher incidence of late (HR 2.3) and major (Clavien–Dindo 3+) (HR 2.1) complications in salvage cystectomy patients, but noted no difference in 5 year disease-specific (63.0% in primary cystectomy versus 63.8% in salvage cystectomy) or 5 year overall survival (47.8% in primary cystectomy versus 29.0% in salvage cystectomy) compared with primary cystectomy patients.^42^

Radiotherapy alone can be used to treat HR-NMIBC, however current data are sparse. A recent systematic review of patients with high-grade T1 (HGT1) NMIBC treated with raditherapy alone showed that 78% of patients experienced complete response, with NMIBC recurrence of 30% and overall invasive-disease progression of only 17%. However, adverse effects in radiation therapy (hemorrhagic cystitis, erectile dysfunction, fecal incontinence, etc.) occur in up to 17% of patients.^43,44^ Emerging retrospective and prospective data such as the RTOG 0926 trial suggest that chemoradiation can provide high bladder-preservation rates (88%) and acceptable oncologic outcomes (3 year OS of 69% and 5 year OS of 56%) for selected patients with recurrent high-grade NMIBC, but randomized evidence directly comparing chemoradiation with RC in NMIBC is lacking.^45^ Additionally, current oncologic outcomes data for TMT are comparable to those for historic salvage intravesical therapies, with above-mentioned next-generation salvage agents becoming more widely used and shown to have superior recurrence-free survival (RFS) and progression-free survival (PFS) outcomes than TMT.^46^ Current guidelines continue to recommend radical cystectomy as the standard for many very high-risk or BCG-unresponsive NMIBC patients. On the basis of current guidelines and expert opinions, the use of TMT should be counseled for use only on-trial for NMIBC.

### Surgical Considerations

Overall, salvage radical cystectomy (SC) seems feasible and does not show any higher rates of perioperative and early (< 90 days) complications compared with primary radical cystectomy (PC) in retrospective studies.^41,42^ The rate of any major late complications such as ureteral stricture (47.1% PC versus 75% RC), bowel obstruction (55.6% PC versus 75% RC), and parastomal hernia (22.2% PC versus 25% SC) were significantly higher (HR 2.3), in the salvage group versus primary cystectomy groups, with an increased likelihood of ICU admission following surgery in salvage patients (OR 2.8). These differences may largely be due to the additional technical challenges that salvage cystectomy present to the surgeon. Adhesions and fibrosis of surrounding tissues from the chemotherapy and radiation regimens can provide challenges in identifying and isolating anatomic structures intraoperatively and provide a hostile abdomen in which the surgeon must work.^47^ Chemoradiation induces tissue changes (fibrosis, friability, etc.) that increases the risk for long-term bowel and ureter complications, such as anastomotic leaks, strictures, and bowel obstructions. Some suggest the use of a more proximal segment of ileum for the urinary diversion, and excision of the distal ureters to try and exclude as much radiated tissue from the anastomoses as possible.^40,48,49^ Additionally, the use of intraoperative indocyanine green (ICG) to assess tissue vascularity has become popular. Literature has shown that ICG use decreases stricture rates, with one study showing zero strictures with use of ICG versus a 10.6% stricture rate without,^50^ and a large-scale Cochrane review of over 1300 patients found significantly decreased odds of stricture (OR 0.2, 95% confidence interval (CI) 0.07–0.52) with ICG use, as well as decreased rate postoperative major complications (OR 0.54, 95% CI 0.35–0.83).^51^

Currently, the role of robotic salvage cystectomy is not defined within the guidelines. There is currently insufficient evidence to suggest one approach versus another for both functional and oncologic outcomes, but it is worth noting that the robotic approach is associated with increased operative time and decreased blood loss, albeit without any differences in long-term outcomes or complications.^52–54^ Future research is warranted on the outcomes and differences between robotic and open approaches for salvage cystectomy.

Aside from TMT, there is exciting work underway examining the use of systemic therapy alone for bladder preservation. The RETAIN-1 trial employed a risk-adapted approach and administered dose-dense methotrexate, vinblastine, doxorubicin (adriamycin), and cisplatin (ddMVAC) neoadjuvant chemotherapy (NAC) to patients with T2–3 MIBC who were then closely surveilled and achieved a 73% 2 year metastatic-free rate with 48% of patients on active surveillance avoiding cystectomy, with a 15% ypT0 rate in patients who underwent cystectomy. Their follow-up study RETAIN-2 employed the same approach but added in immunotherapy with nivolumab to the NAC regimen, and employed the use of tumor next-generation sequencing (CARIS) to assess for tumor mutations. Those patients with a tumor mutation and no evidence of disease after initial TURBT were then given NAC+nivolumab and enrolled in active surveillance. Results were encouraging, with an improved 78% metastasis-free survival (MFS) rate in patients on active surveillance patients, as well as an increased ypT0 rate of 40% in those undergoing cystectomy, suggesting an additive benefit of adding nivolumab to ddMVAC.^55,56^

## Future Directions

Continued innovation is essential to improve bladder-sparing strategies for patients with high-grade NMIBC who are unwilling or unable to undergo radical cystectomy. Several emerging developments have the potential to refine patient selection, enhance treatment response assessment, and support durable bladder preservation.

A major limitation of current approaches to bladder sparing is the challenge of determining true disease clearance. Standard clinical complete response criteria rely on cystoscopy, urine cytology, and resection-based sampling, all of which may fail to detect residual or occult disease. Molecular response assessment is an area of growing interest. Early data suggest that longitudinal monitoring of urinary tumor DNA (utDNA) may correlate with sustained response to intravesical salvage therapy, while the persistent absence of circulating tumor DNA (ctDNA) may indicate disease confined to the bladder.^57,58^ Rising levels of utDNA or ctDNA could help flag early molecular recurrence, prompting earlier response assessment such as repeat TURBT or interval imaging and potentially expediting intensified intravesical therapy or timely salvage surgery.

Predicting response to intravesical therapies remains another challenge. Artificial intelligence (AI)-based biomarkers are being developed to integrate clinical variables, cystoscopic features, and histopathologic data to improve treatment selection. The Valar lab Vesta biomarker is an example of a predictive platform that can estimate the likelihood of BCG response.^59^ As the intravesical treatment landscape expands, patients may benefit from additional AI-based predictors tailored to newer therapeutic classes. Preclinical systems that use primary tissues, including patient-derived tumoroid cultures, represent another emerging strategy.^60^ These platforms may allow in vitro testing of drug sensitivity before exposure to patients and possibly help guide individualized therapy selection.

Further validation is necessary before these technologies can be incorporated into routine clinical practice, but together they represent promising avenues toward a more precise and durable approach to bladder preservation in high-risk NMIBC.

## Conclusions

In patients with BCG-unresponsive NMIBC, early radical cystectomy remains the most reliable curative option for those who are surgically fit, while intravesical or systemic bladder-sparing therapies may be considered when surgery is declined or contraindicated, provided that strict surveillance and readiness for salvage surgery are maintained. Following trimodal therapy for MIBC, non-muscle-invasive relapses can often be managed per NMIBC pathways, but muscle-invasive recurrences demand prompt salvage cystectomy, where outcomes can approximate those of primary surgery if performed without delay. Across both settings, rapid workup, multidisciplinary coordination, strict surveillance, and shared decision-making are essential to balance oncologic safety with patient quality of life.
